# News Items

**Published:** 1917-02

**Authors:** 


### News Items.

---

Dr. Q. C. Gaithes, of Glen Rose, has recently been in New Orleans doing post-graduate work.

---

Drs. F. C. Beall, Clay and Johnson, of Fort Worth, have let the contract for the erection of a sanitarium.

---

The American Public Health Association and the *American Journal of Public Health,* 755 Boylston Street, Boston, Mass., is now located at 126 Massachusetts Avenue, Boston, Mass.

---

Dr. Philip Skrainka, of St. Louis, Mo., has severed his connection as literary editor with the *Interstate Medical Journal,* and will in this month start a journal of his own, which will be known as *Medicine and Surgery.* We wish for Dr. Skrainka great success.

